# Report of similar placebo response in one internet versus onsite randomised controlled trials from the literature

**DOI:** 10.1016/j.ocarto.2024.100474

**Published:** 2024-04-27

**Authors:** Arthur Ooghe, Xiaoqian Liu, Sarah Robbins, Jillian P. Eyles, Leticia A. Deveza, Samuel Branders, Frédéric Clermont, Alvaro Pereira, David J. Hunter

**Affiliations:** a Cognivia, 11 rue Granbonpré, Bte 9 - 1435 Mont-Saint-Guibert, Belgium; b Rheumatology Department, Royal North Shore Hospital, Northern Clinical School, Faculty of Medicine and Health, The University of Sydney, Australia; c Sydney Musculoskeletal Health, Kolling Institute, The University of Sydney, Australia

**Keywords:** Placebo response, Remote trial, Osteoarthritis, Analgesia, Patient-physician interaction

## Abstract

**Objective:**

The aim of this study was to compare the magnitude and the predictors of the placebo response in an internet versus onsite randomised controlled trials (RCTs) in people with hand osteoarthritis (HOA).

**Method:**

This study is a *post-hoc* analysis based on one internet RCT (RADIANT) and previously published onsite RCTs for HOA identified through a rigorous searching and selection strategy. The magnitude of the placebo response in the two different types of RCTs were compared using heterogeneity statistics and forest plots visualisation. Classic placebo predictors as well as a combined model, defined with data from onsite RCTs, were tested to predict the placebo response.

**Results:**

We analysed the dataset from RADIANT and fourteen previously published onsite RCTs. None of the analyses showed a significant difference between the placebo response for the internet versus onsite RCTs. The “classic” placebo predictors combined in a multivariate predictive model correlated significantly with the placebo response measured in RADIANT study.

**Conclusion:**

Despite the absence of face-to-face interactions with the study personnel, there is no evidence that either the magnitude or the predictors of the placebo response of this internet RCT differ from those of onsite RCTs. This analysis is considered as a first step towards evaluating the difference between these designs and strengthens the argument that internet RCTs remain an acceptable alternative way to assess the efficacy of an active treatment in comparison to a placebo.

## 1 Introduction

The COVID-19 pandemic has highlighted some limitations of classic ran-domized controlled trial (RCT) design, including the need to assess and treat participants in-person in the clinic. One solution is to design randomised controlled trials (RCTs) that minimise the need for face-to-face contact, with remote (online) end-point assessment via internet-based questionnaires and surveys.

Interest in using the internet for clinical trials has grown since the 1980s [[1], [2], [3]] but the internet has played a more significant role since the beginning of the 21st century, through the development of remote clinical trials [4]. Until now, the examples of the use of online trials are mainly trials designed for cognitive behavioral therapies [5,6], such as behavioral therapies for depression [7] to help patients cope with their pain [8,9], or to remotely deliver physiotherapy advice and protocols to patients for their exercise regimens [9,10,11]. Conversely papers reporting online trials to assess the efficacy of analgesic drugs are rare (e.g.,[12]), especially with designs involving a placebo-control group. Internet RCTs are in their infancy, and little is known about how they perform compared with classic onsite RCTs. In particular, the characteristics and magnitude of the placebo response for internet RCTs have rarely been assessed.

Studying the placebo response is highly important in drug development, especially for analgesic drugs, as randomised placebo-controlled trials are the gold standard. It has become even more important, considering that its magnitude in analgesia RCTs has tended to increase with the year of trial completion [13]. A recent meta-analysis of the control group treatments in hand osteoarthritis (HOA) showed that placebo response was highly significant in this indication [14]. In particular, it suggested that HOA pain showed a greater benefit from placebo treatment compared to knee OA.

Despite that placebo effect is a well-studied mechanism, there has been much controversy over the meaning of the word ‘placebo’, as stated by Doherty et al. [15]. It is therefore important to distinguish placebo response from the placebo effect. In the context of a clinical trial, and in particular in this paper, the placebo response could be described as the improvement in symptoms in participants assigned to a placebo treatment. This could encapsulate various mechanisms such as the natural course of the disease, the Hawthorne effect, the regression-to-the-mean, and the placebo effect itself.

As the placebo response is often described as a function of the relation-ship between investigators and the patients [16,17], this placebo response could be lower in an online design due to the lack of face-to-face interactions. Although a previous study, exploring the placebo analgesic response on healthy patients experimenting pain through heat stimulations, showed that a placebo effect can be elicited with information delivered online [18], this study could not determine if this internet placebo effect was more or less powerful than traditional face-to-face interactions. Moreover, there has been no study evaluating the difference between the placebo response observed in internet RCTs and classic onsite RCTs to the best of our knowledge. Our first aim was therefore to leverage data from a recent online RCT to assess if the observed placebo response in this internet RCT differ from the magnitude of a study with a classic onsite design.

Furthermore, this absence of face-to-face interactions with investigators may impact the mechanisms of the placebo response in an internet RCT. Psychological and neurobiological mechanisms of placebo analgesia have been extensively studied in the literature [19,20]. Links have been made to personality traits [[19], [20], [21]] and expectations for improvement [[22], [23], [24]]. Patient disease intensity measures [25] and demographics [26,27] are other well studied placebo predictors in classic RCTs. With this study, we aimed to assess the pertinence of these predictors in an internet RCT and if the underlying mechanisms of the placebo analgesia remain similar.

This analysis is therefore the first step to assess the comparability in the results of internet RCT and those of RCTs with classic designs. Furthermore, better understanding the placebo response in internet RCTs would help to properly define the study design by allowing for more precise sample size estimates and to identify potential prognostic factors which could be used in adjusted analysis to increase the precision of the measurement of the treatment response [28].

## 2 Method

### 2.1 Study data used in the analysis

**Fig. 1:**
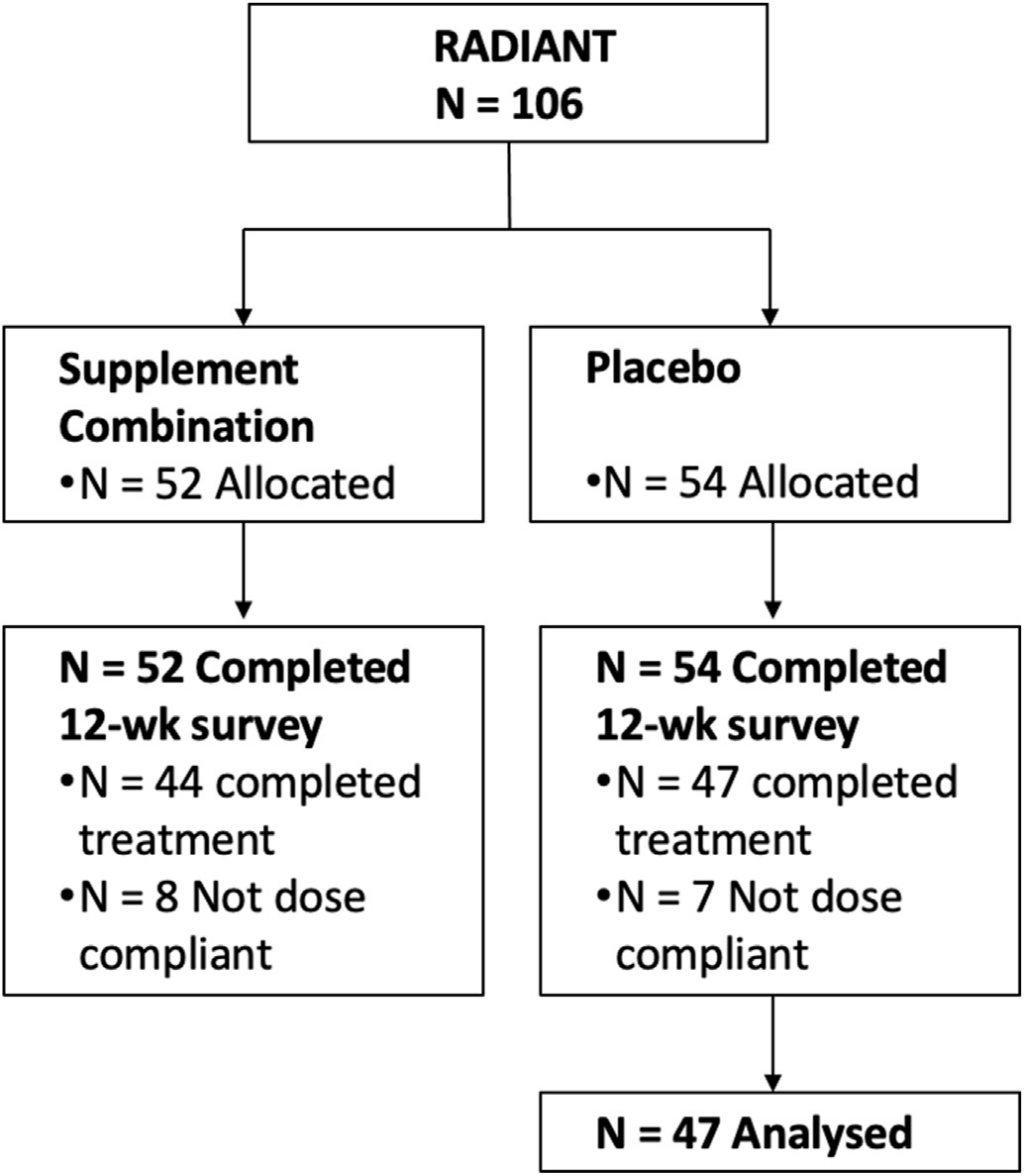
Analysed population from RADIANT.

This *post-hoc* analysis was based on the clinical data from a recently published online RCT investigating the efficacy and safety of a supplement combination in hand OA (the RADIANT study) [29]. One hundred and six participants with clinical and radiographic evidence of hand OA were randomly assigned to take orally either a supplement combination composed of [1]: combined supplement containing Boswellia serrata extract, pine bark extract and methylsulfonylmethane and [2] curcumin or placebo twice a day for 12 weeks. All the participants were aged 40 years and over and had hand pain exceeding 40 out of 100 on a Visual Analogue Scale (VAS) and functional disability 6 and over out of 30 using Functional Index of Hand Osteoarthritis (FIHOA). The outcome measures included self-reported pain using VAS, Patient Global Assessment (PGA), and FIHOA using an online survey. The findings of the RADIANT study indicated that there was no statistically significant difference between the responses measured in the intervention and placebo arms [29]. This *post-hoc* analysis included the 47 participants of the placebo group (see Fig. 1).

The magnitude of the placebo response measured in the RADIANT study was compared with other previously published classic studies with similar control groups and administration methods. These studies published were identified by literature review using keywords [“HOA” OR “hand osteo-arthritis” OR (“osteoarthritis of the hand”) OR (“osteoarthritis of the hands”)] in the database of PubMed. The selection criteria were: i) oral treatment, ii) placebo controlled RCTs for HOA, iii) at least one of the following outcome measures was assessed: pain VAS 0–100, Numerical Rating Scale (NRS) 0–10, FIHOA 0–30, or PGA 0–100. Considering that the placebo response has considerably increased recently over time [30], only the results published in the last 20 years were used in this analysis.

The selection scheme with the number of excluded papers for each criterion can be found in supplementary materials. The measurement of pain using a NRS (0-10) was rescaled on a scale from 0 to 100 to be compared with the VAS scores of the other studies. Both measures are simply named as Average Pain Score (APS) below.

### 2.2 Statistical analysis for the magnitude of the placebo response

The comparison between the placebo response measured in RADIANT and in the classic studies found in the literature was assessed, for each endpoint, by the heterogeneity of the measured placebo response. Based on the subgroups internet (RADIANT) vs. classic design (data from literature), the Cochrane's heterogeneity statistic Q was computed for the differences between and within the subgroups. The heterogeneity I^2^ statistic was also computed for the whole set of studies and subgroups. This statistic was evaluated using the considerations of Higgins et al. [31]: mild heterogeneity going from 0% to 31%, moderate heterogeneity going from 31% to 56% and strong heterogeneity going from 56% to 100%. All the heterogeneity statistics computations were performed and plotted using the R package metamean [32]. At last, a *χ*^2^ test for subgroup differences was performed based on the difference in design.

As the baseline value of the endpoints may influence the placebo response [25], the Cochrane's heterogeneity statistic Q was also computed based on subgroups defined by the mean baseline value of the efficacy measures in each study. The subgroups were defined following the values reported in the selected studies.

### 2.3 Analysis on the placebo predictors

Baseline disease intensity measures [25] and expectations [22,23] are two of the features that are the most commonly presented as placebo predictors in the literature. The univariate correlations of the placebo response with three features were therefore evaluated:

• the baseline value of the endpoint,
• the number of painful joints
• the expectations of relief, evaluated with questions such as “I think I will feel better at the end of this study treatment “on a five-point NRS.

Nevertheless, a lot of other baseline patients' factors and features are described in the literature as predictors of the subjects’ placebo response such as:

• Medical History [33].
• Demographics (age, sex, Body Mass Index (BMI), etc) [26,27,33],
• Patient psychological characteristics (or traits) linked to the placebo response [[19], [20], [21], [22], [23], [24]].

All these factors are partly correlated and have an entangled impact on the placebo response. To account for the multivariate nature of the classic predictors, we measured their combined predictive power inside a prespecified prognostic multivariate model, Placebell [34]. This multivariate model was developed on data from analgesia RCTs with classic onsite designs using machine learning methods. This multivariate approach was predictive of the placebo response in classic RCTs and in particular, in knee/hip OA with Pearson's correlations between 0.45 and 0.60, depending on the endpoints [35].

The Pearson's correlation between the placebo response as measured by the FIHOA in RADIANT study and the prognostic multivariate model was calculated to assess the performance of the classic predictors. This analysis was restricted to the FIHOA which was considered as the endpoint with the most qualitative data. Indeed, since the error in assessing efficacy can be viewed as normally distributed around zero, it should not affect the placebo response when averaged across the population. Conversely, given that correlations assess differences between individuals, excessive noise in the measurements could hinder the evaluation of these correlations. This consideration, based on previous developments [36], is assessed in the supplementary materials.

## 3 Results

### 3.1 Charaterization of the data collected for the comparison with RADIANT data

The whole dataset access was achieved for the RADIANT study. Fourteen previously published onsite studies were identified from literature searching [[37], [38], [39], [40], [41], [42], [43], [44], [45], [46], [47], [48], [49], [50]]. The studies found were consistent with the studies reported in a recent meta-analysis [14] which were compliant with our criteria. For these studies, the analysis was based on the published data. Eleven of the fourteen studies had an APS measured using a VAS or an NRS, 6 had a measurement of PGA and 5 had a measurement of FIHOA. The baseline patients’ characteristics of these studies can be found in Table 1.

**Table 1:** Baseline characteristics of the studies used in the analysis.

	N	Mean Age (SD), years	N females (%)	Mean BMI (SD), kg/m^2^	Mean APS (SD)	Mean FIHOA (SD)	Mean PGA (SD)
Grifka et al., 2004	196	62.7 (11.7)	162 (82.7)	27 (4.8)	71.2 (12.6)	–	62.1 (16)
Kvien et al., 2007	41	59.6 (5.3)	38 (93)	–	62.1 (16.9)	–	62.3 (17.9)
Gabay et al., 2011	82	63 (7.2)	62 (75.61)	25 (3.9)	53.6 (14.2)	10.3 (3.8)	–
Wenham et al., 2012	35	61.1 (9)	31 (89)	27.7 (5.4)	58 (17)	–	59 (18)
Shin et al., 2013	44	58.6 (7)	42 (95.5)	24.7 (2.7)	–	5.3 (4.9)	60.9 (18.9)
Park et al., 2016	106	59.4 (8)	98 (92.5)	23.9 (2.8)	–	–	50.1 (16.3)
Sofat et al., 2017	22	62.4 (8.7)	19 (86.4)	27 (4.3)	64 (14)	–	–
Kingsbury et al., 2018	119	62.6 (9.1)	102 (86)	29.4 (6.3)	68 (18)	–	–
Lee et al., 2018	98	58.3 (7)	82 (84)	–	44.9 (22.9)	–	–
Kroon et al., 2019	46	65.6 (8.5)	35 (76)	27.2 (4.9)	35.4 (28.5)	11 (4.7)	55.7 (22)
Davis et al., 2021	32	66 (7)	27 (84)	29.3 (6)	64 (17)	–	–
Ferrero et al., 2021	32	67.5 (8)	29 (91)	24.6 (4)	63.9 (16)	12.6 (5)	–
Vela et al., 2022	66	61.5 (23.94)	46 (70)	26.25 (9.02)	61 (40.87)	–	–
Williams et al., 2022	14	58.3 (3.4)	14 (100)	24.3 (3)	–	6.4 (5.5)	–
RADIANT	47	66.34 (7.81)	35 (74.47)	27.66 (6.1)	61.15 (12.14)	10.62 (3.88)	43.98 (18.62)

As presented in Table 1, the patients demographics in RADIANT (internet RCT) and the 14 onsite studies were mainly similar: mean age around 60–65 years, predominantly females (70%–100%), who were overweight on average (BMI from 23.9 to 29.4 ​kg ​m^*−2*^). On the contrary, there was a large difference between the baseline efficacy measurements. Considering the APS, most of the studies had a mean baseline value around 60 (53.6–68), but two studies had a value under 50 [45,46] and one had a value over 70 [37]. All the studies had a mean baseline value of FIHOA between 10 and 13, but two had a value approximatively two times lower [41,50]. The average baseline values of the PGA were mostly between 55 and 65, while they were lower for RADIANT and one onsite study [42].

The placebo response measured in RADIANT and the previously published onsite studies is presented in Table 2. A high correlation was found between the mean placebo response measured with the APS and the mean baseline value of the APS (r ​= ​0.79, 95% CI ​= ​[0.40, 0.94], p ​= ​0.002). This supported the consideration of the difference in baseline values in the heterogeneity analysis. The correlations between the placebo response measured by the PGA and the FIHOA and their respective baseline values were also positive but not significant (0.30 and 0.45, respectively).

**Table 2:** Mean(SD) of the Placebo Response measured with Average Pain Score (APS), Functional Index of Hand Osteoarthritis (FIHOA) and Patient Global Assessment (PGA).

	APS	FIHOA	PGA
Grifka et al., 2004 (N ​= ​196)	19.3 (20)	–	9.4 (20)
Kvien et al., 2007 (N ​= ​41)	6.3 (21.13)	–	4.2 (21.13)
Gabay et al., 2011 (N ​= ​82)	11.3 (24)	0.7 (4.8)	–
Wenham et al., 2012 (N ​= ​35)	16 (18.92)	–	15 (20.38)
Shin et al., 2013 (N ​= ​35)	–	−0.6 (4)	12.7 (24.6)
Park et al., 2016 (N = NA)	–	–	6 (40.47)
Sofat et al., 2017 (N ​= ​19)	9 (25.93)	–	–
Kingsbury et al., 2018 (N ​= ​119)	13.1 (27.3)	–	–
Lee et al., 2018 (N ​= ​98)	−0.4 (25.89)	–	–
Kroon et al., 2019 (N ​= ​46)	−0.2 (17.3)	0.5 (4)	8 (25.3)
Davis et al., 2021 (N ​= ​32)	18 (23.03)	–	–
Ferrero et al., 2021 (N ​= ​32)	11.7 (24)	0.2 (5)	–
Vela et al., 2022 (N ​= ​66)	11.4 (26.73)	–	–
Williams et al., 2022 (N ​= ​14)	–	0.7 (6.09)	–
RADIANT (N ​= ​47)	16.4 (23.9)	1.4 (3.85)	7.3 (29.58)

### 3.2 Comparison of the magnitude of the placebo responses

**Fig. 2:**
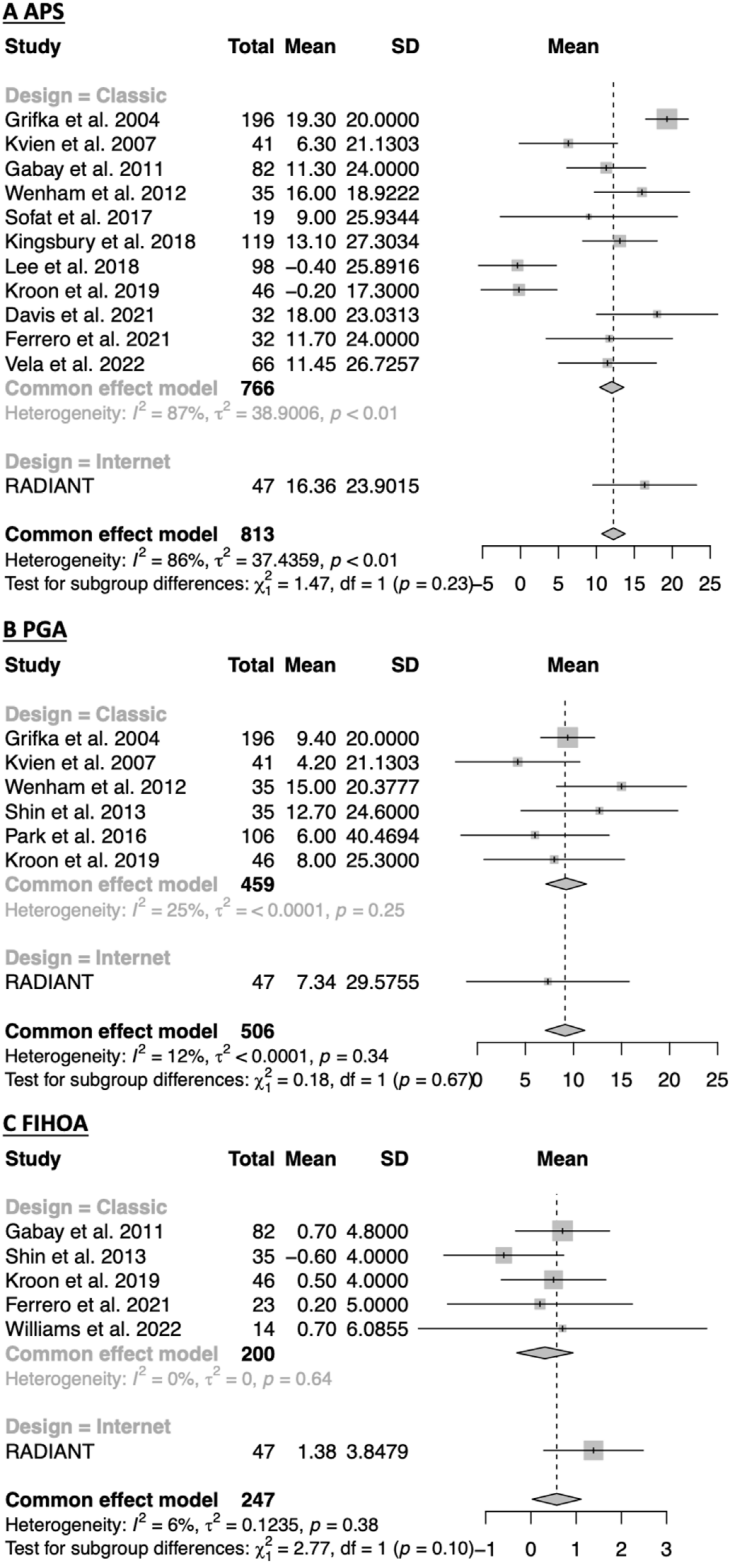
Comparison of the placebo responses measured in RADIANT and in other papers from the literature. Study ​= ​Name of the study, Total ​= ​Number of subjects, Mean ​= ​Mean of the placebo response, SD = Standard deviation of the placebo response, p ​= ​p-value of the test with the null hypothesis I^2^ ​= ​0.

The comparison of the placebo response measured in the RADIANT study with 14 previously published onsite studies is presented in Fig. 2. All the computed measures of heterogeneity are presented in Table 3.

**Table 3:** Heterogeneity statistics for the considered endpoints. Significance levels for the Cochrane's Q statistic: *∗*: p ​< ​0.05, *∗∗*: p ​< ​0.01, *∗∗∗:* p ​< ​0.001.

	I^2^ Statistic Estimate CI 95%	Cochrane's Q Statistic				
		Total	Comparison internet vs. Classic Design		Comparison High vs. Low Baseline	
			Between-groups	Within groups	Between-groups	Within groups
APS	86% [78%,92%]	80.2*∗∗∗*	1.5	78.7*∗∗∗*	71.7*∗∗∗*	8.5
PGA	12% [0%,74%]	6.8	0.2	6.6	0.9	5.9
FIHOA	6% [0%,76%]	5.3	2.8	2.5	3.1	2.2

For the APS, the heterogeneity statistic I^2^ was strong (86%) and significant. Nevertheless, the *χ*^2^ test for subgroup differences based on the design was not significant (p ​= ​0.23). This test result is reinforced with the analysis of the Cochrane's Q statistics (presented in Table 3). This showed that the heterogeneity was not due to the differences of study design (Q ​= ​1.5) but to the variability of the responses measured in onsite studies (Q ​= ​78.7, p ​< ​0.001). As introduced in the previous section, a large difference between the average baseline APS of the studies was observed. The analysis con-firmed then that the placebo response heterogeneity came mainly from the difference of APS at baseline (Q ​= ​71.7; p ​< ​0.001). Therefore, there was no significant difference of placebo response for studies with similar average baseline APS, no matter their design (Q ​= ​8.5). In particular, the I^2^ statistic was very low (6%) for the group with a “medium” APS baseline value (composed by 8 onsite studies and RADIANT). The influence of the baseline value on the heterogeneity of placebo response is also discussed in Supplementary Material 3.

For the PGA, the heterogeneity statistic I^2^ was mild (12%). The *χ*^2^ test for subgroup differences based on the design was not significant (p ​= ​0.67). As presented in Table 3, no Q statistic was significant. Nevertheless, the heterogeneity came more from the differences between studies with a different PGA at baseline (Q ​= ​0.9), than from the differences of study design (Q ​= ​0.2).

For the FIHOA, the heterogeneity statistic I^2^ was mild (6%). The χ2 test for subgroup differences based on the design was not significant (p ​= ​0.10). As for the PGA, no Q statistic was significant, but the heterogeneity came also more from the differences between studies with a different FIHOA at baseline (Q ​= ​3.1), than from the differences of study design (Q ​= ​2.8). None of these results showed a statistically significant heterogeneity coming from the differences between the internet and onsite studies. Altogether, this suggests that the heterogeneity in the measured placebo response was mainly due to differences in the mean baseline efficacy value of the studies and not in their design.

### 3.3 Placebo prediction for the internet RCT

The baseline value of the FIHOA, the number of painful joints, and the expectations were, as described in the literature, positively correlated with the placebo response, as measured by the FIHOA endpoint in the RADIANT study. These correlations were 0.26 (p ​= ​0.08), 0.15 (p ​= ​0.32), and 0.22 (p ​= ​0.14) for the FIHOA, the number of painful joints, and the expectations, respectively. On a multivariate level, the combination of predictors inside the prognostic multivariate model had a statistically significant correlation of 0.38 (95% CI: [0.19, 0.54], p ​< ​0.001) with the placebo response as measured by the FIHOA endpoint. The model using standard predictors, learned from data of onsite RCTs, was then applicable to this internet RCT.

## 4 Discussion and conclusion

The aim of this *post-hoc* analysis was to assess potential differences of the placebo response between an online study and a group of onsite previously published studies in people with HOA investigating oral analgesics and supplements.

First, we compared the magnitude of the placebo response measured in the RADIANT study, the internet RCT, to the placebo response measured in other HOA studies with classic design. This *post-hoc* analysis did not show that the change in study design brought significant differences in the placebo response. Namely, the *χ*^2^ tests showed that the placebo response, as measured by the APS, the PGA, or the FIHOA, was not significantly different between RADIANT and the onsite studies. Furthermore, the heterogeneity of the placebo response among the different studies appeared to come mainly and significantly from the differences between the mean baseline values of the efficacy measures. This was confirmed by a high correlation observed between the mean baseline APS of the studies and their placebo response as measured by the APS. This last observation is in accordance with the literature results [25]. Overall, despite the absence of face-to-face interactions with study personnel, the magnitude of the placebo response in this internet RCT was not different to that measured in the onsite RCTs.

Secondly, we investigated the consistency of classic placebo predictors in the RADIANT study. The correlations of the baseline efficacy measurement, the number of painful joints and the expectations were positively correlated with the placebo response, as in onsite studies, according to the literature [22,23,25]. However, these correlations were not significant. The absence of significance may be caused by the limited sample size used. The placebo response is a complex and multivariate mechanism. Therefore, a prespecified prognostic multivariate model, Placebell, was proposed as an integrative model to focus on multidimensional mechanisms that can trigger placebo response in a clinical trial setting. The model was trained to predict the placebo response on data from analgesia RCTs with onsite design [34]. This multivariate combination of baseline placebo predictors had a statistically significant correlation of 0.38 (p ​< ​0.001) with the placebo response as measured by the FIHOA endpoint. This correlation was slightly lower than in a previous onsite study [35] that showed values between 0.45 and 0.60. However, this difference might be explained by the correlation variability, considering the measured confidence intervals of the correlation in RADIANT study (95% CI: [0.19*,* 0.54]).

As the difference in study design (online or onsite) was not a large contributor, in this analysis, to the heterogeneity of the measured placebo response, this consistency in the tested placebo predictors aligns with the hypothesis that the main mechanisms causing the placebo response are consistent across the two trial settings. Overall, the study design (onsite or online) appears, in this analysis, to play a much smaller role in the placebo response compared to other factors already discussed in the literature, particularly the baseline pain value.

There are a few limitations of this *post-hoc* analysis. First, while the original data of the RADIANT study were available to perform the analysis, only published data were available for the previously reported onsite studies. The same metrics were therefore not always provided in the published data. Second, there were only a few studies reporting a placebo response of an oral treatment for HOA as measured by the change from baseline of the PGA or the FIHOA. The comparisons performed for these endpoints should therefore be taken with caution. Third, as with most systematic reviews, there is potential for publication bias, which results in finding more trials with “positive” outcomes than trials that have failed to demonstrate statistical superiority of their treatment compared with the placebo. Finally, the comparison between the study design was performed using only one online study, RADIANT, as we found no other online RCT for HOA published in the literature. Nevertheless, the results of this first analysis comparing the placebo response in an Internet study with onsite studies are encouraging as there is no evidence that either the magnitude or the predictors of the placebo response differ between an internet RCT and the onsite RCTs. On the contrary, the response measured in RADIANT was closed to the response measured in the onsite studies with similar baseline values and the prognostic model designed for onsite studies was significantly associated with this online placebo response. However, as more data of online clinical trials become available further analyses should be performed to confirm these conclusions on HOA and on other pain-related diseases.

If confirmed, assuming the absence of large impact of the design on the placebo response would provide some evidence that internet RCTs should be considered as a reliable method to assess the efficacy of an active treatment in comparison to a placebo (if the proposed safety profile of the therapy allows for such a design). Furthermore, if the placebo response is proved to be similar whether the study is conducted onsite or remotely, the possibility would exist to construct hybrids of the two designs. This would potentially facilitate participant recruitment and enable more efficient study conduct. Given the contribution from placebo response to outcomes variability, it remains important to analyze this in the future and to capture this in clinical trials to better understand treatment responses.

## Contributions

DJH and XL conceived of the RADIANT study. XL, SR, LD, JE, and DJH initiated the study design and implementation. SB, FC, and AP designed and implemented the requirements of the Placebell covariate to the RADIANT study. FC followed the implementation of these requirements. AO performed this statistical analysis. SB contributed to the statistical expertise. AO, SB, AP, and XL drafted this article. All authors contributed to the refinement of this article and approved this final version.

## Funding

The RADIANT study was supported by 10.13039/501100000925National Health and Medical Research Council (10.13039/501100000925NHMRC) Program Grant (grant number APP1091302) and by the Lincoln Centre for Bone and Joint Diseases. Cognivia employees (AO, SB, FC, and AP) and their analysis were supported by Cognivia.

## Conflicts of interest

DJH is supported by an 10.13039/501100000925NHMRC Practitioner Fellowship and provides consulting advice for 10.13039/100004334Merck Serono, TLC Bio, 10.13039/100004336Novartis, Tissuegene and 10.13039/100004319Pfizer. JE is a Sydney Health Partners Research Translation Fellow.
