# Supplementary material for: Report of similar placebo response in one internet versus onsite randomised controlled trials from the literature
Source: Osteoarthr Cartil Open. 2024 Apr 27;6(2):100474. doi: 10.1016/j.ocarto.2024.100474 (PMC11088186; doi:10.1016/j.ocarto.2024.100474)
Supplement: Multimedia component 1 [file mmc1.docx]

Supplementary Material – 1

Selection of previously published classic studies

The analysis presented in this paper, used published RCTs investigating the efficacy and safety of other oral therapies for hand osteoarthritis (HOA) in the last 20 years.

To find sources, the research ["hand osteoarthritis" OR "hand osteo-arthritis" OR ("osteoarthritis of the hand") OR ("osteoarthritis of the hands")] was performed on PubMed, selecting all the papers from 2003 to 2022 with an available abstract. This research was performed on the 30th of January 2023 and provided 832 papers.

The following selection criteria were then applied:

1. Was this paper presenting results from randomized trials for HOA, excluding RADIANT and COMBO studies? (Yes/No);
2. If yes, was the study placebo controlled? (Yes/No);
3. If yes, was it a study on an oral treatment? (Yes/No);
4. If yes, was there, at least, an assessment of one of the following measurements:
   1. Pain - Visual Analogue Scale (VAS) 0-100,
   2. Pain - Numerical Rating Scale (NRS) 0-10,
   3. Functional Index of Hand Osteoarthritis (FIHOA) 0-30,
   4. Patient Global Assessment (PGA) VAS 0-100

Were these measurements reported and usable?

The selection scheme and the number of considered papers can be found in Figure 1. Eleven papers were therefore used for the analysis of the APS, five for the analysis of the FIHOA, and six for the analysis of the PGA.


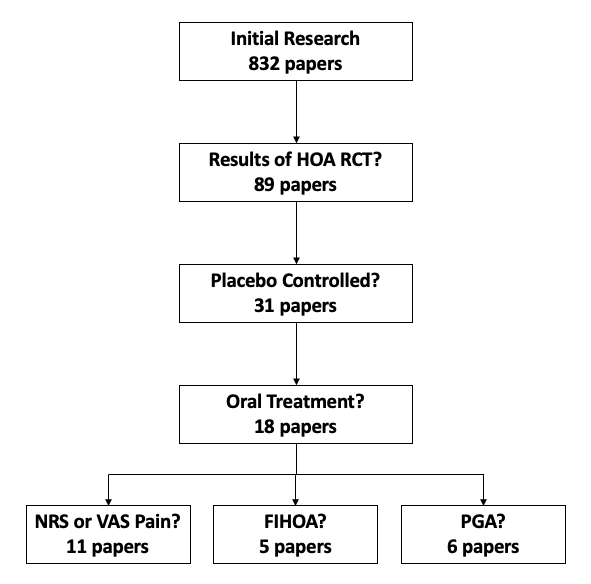


Figure 1:Flow diagram of study selection.
