# Supplementary material for: Report of similar placebo response in one internet versus onsite randomised controlled trials from the literature
Source: Osteoarthr Cartil Open. 2024 Apr 27;6(2):100474. doi: 10.1016/j.ocarto.2024.100474 (PMC11088186; doi:10.1016/j.ocarto.2024.100474)
Supplement: Multimedia component 2 [file mmc2.docx]

# Supplementary Material – 2

# Analysis of the Evaluation Error in the RADIANT study

## 1 Introduction

This analysis was based on an abstract presented in the OARSI Congress (1). It intended to estimate the pain evaluation variability and its evolution over time during the course of a clinical trial. Indeed, the intrinsic subjectivity of pain may render the evaluation of the pain-related questionnaires difficult. A high consistency in the subjects’ pain ratings could therefore help to increase the quality of study data.

Here, we used the formula presented in this abstract to evaluate the quality of the data of each endpoint assessed in the RADIANT study.

## 2 Method

To study this inconsistency in the pain evaluation, the measured pain scores, for example here the Average Pain Score (APS), was modeled as the sum of:

- A signal, which represents the “ideal” measure that the patient would have reported if he/she was totally consistent
- An evaluation error ε, disrupting the consistent and ideal value of APS


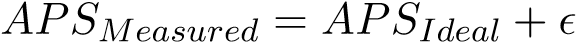


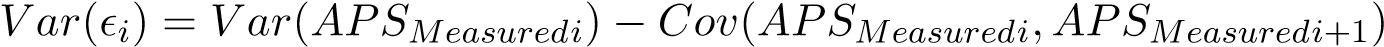
Based on that, the importance of the evaluation error could be estimated using an estimator of its variance defined by:

where ε *_i_* is the error at visit i, and *APS_i_* is the APS recorded at visit i. The higher the variance of the error is, the worse the quality of the measurements would be.

Nevertheless, it could be more interesting to compare this variance with the total measured variance. Indeed, if we measure the *δ* ratio defined as


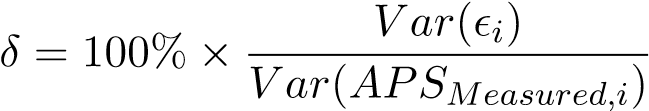


it could provide information about the level of certainty we could have by comparing the values reported by different subjects or populations.

We used this formula to compute the *δ* ratio in RADIANT to compare the quality of the different efficacy measures at Baseline, Week 2, and Week 6.

## 3 Results

The results of the computed *δ* ratio are presented in Table 1.

|  | APS | FIHOA | PGA |
| --- | --- | --- | --- |
| Baseline | 45.86% | 17.04% | 76.74% |
| W2 | 44.43% | 22.42% | 59.28% |
| W6 | 42.48% | 23.71% | 50.78% |

Table 1: *δ* ratio for the three efficacy measures in RADIANT.

## 4 Conclusion

Based on the results presented above, the FIHOA seemed to have been a more stable and qualitative measure in RADIANT study. This could be related to the fact that the FIHOA is the sum of several items, reducing the impact of the error of each item on the reported total score.

## References

[1] Ooghe A, Branders S, Pereira A. Can daily self-assessment induce a learning effect mitigating pain evaluation error in clinical trials? Osteoarthritis and Cartilage. 2021 apr;29:S262-3. Available from: [https://linkinghub.elsevier.com/retrieve/pii/S1063458421003836.](https://linkinghub.elsevier.com/retrieve/pii/S1063458421003836)
