# Supplementary material for: Report of similar placebo response in one internet versus onsite randomised controlled trials from the literature
Source: Osteoarthr Cartil Open. 2024 Apr 27;6(2):100474. doi: 10.1016/j.ocarto.2024.100474 (PMC11088186; doi:10.1016/j.ocarto.2024.100474)
Supplement: Multimedia component 3 [file mmc3.docx]

# Supplementary Material – 3

# Analysis of the Placebo Response accounting for baseline heterogeneity

This paper analysed the heterogeneity of the placebo response in study assessing oral treatment for hand osteoarthritis. This supplementary material presents in more detail the results an figures associated to this analysis while accounting for the heterogeneity of the reported symptoms at baseline.

First the correlation was directly evaluate between the average placebo response of each study and the associated average baseline measurement. The following Pearson’scorrelations were observed:

- APS: r = 0.79 (p = 0.002, 95% CI = [0.40;0.94]), evaluated with 12 studies
- PGA: r = 0.30 (p = 0.52, 95% CI = [-0.59;0.86]), evaluated with 7 studies
- FIHOA: r = 0.45 (p = 0.38, 95% CI = [-0.57;0.92]), evaluated with 6 studies

These correlations are always positive but only the correlation of the APS response with the APS baseline was significant due to a lack of available date and statistical power for the two other measurements.

Additionnally, a subgroup analysis of the heterogeneity of the placebo response by defining the subgroup as followed:

- APS:
  - Low: APS Baseline < 50
  - Medium: APS Baseline in [50, 70]
  - High: APS Baseline > 70
- PGA:
  - Low: PGA Baseline < 55
  - High: PGA Baseline > 55
- FIHOA:
  - Low: FIHOA Baseline <10
  - High: FIHOA Baseline >10

The results for APS, PGA, and FIHOA are presented, respectively, in Figure 1, Figure 2, and Figure 3. The placebo response average for each group follows the expected trend: low for the low baseline groups and high for the high baseline groups. Additionally, no within-subgroup heterogeneity was significant. However, the medium group of the APS is the only were the statistical power can be considered as sufficient to detect the presence of a moderate heterogeneity. In this specific group, that includes the RADIANT study, the I^2^ heterogeneity statistics was really low, with a value equal to 6%.

A last analysis, not presented in the associated paper, considered the APS baseline value as an adjusting covariate in a meta-regression. This allows to evaluate the effect of the APS baseline measurement on the heterogeneity of the APS response while considering the data of all the studies (12). This meta-regression was performed with the package Metafor in R. Accounting for the difference in average baseline APS in the analysis of the heterogeneity showed that 84.16% of this heterogeneity can be explained by the difference in baseline value (p < 0.001). Accounting for this baseline difference, the heterogeneity is no longer significant among the 12 studies (Q = 16.32, I^2^ = 40%, p = 0.09).

Overall, these analyses show that a large majority of the heterogeneity of the placebo response can be attributed to the heterogeity in baseline values of the symptoms. This important role of the baseline value is consitant with the literature (e.g. a recent meta-analysis stating that setting a minimum of the baseline value will increase the placebo response [1]). These analyses are limited to the scope of this meta-analysis: the placebo response measured in randomised placebo-controlled trials evaluating the improvement in symptoms of subjects suffering from hand osteoarthritis treated orally.

Reference:

1. Nakagawa Y, Tatebe M, Yamamoto M, Kurimoto S, Iwatsuki K, Hirata H. Choice of control group treatments in hand osteoarthritis trials: A systematic review and meta-analysis. Seminars in Arthritis and Rheumatism. 2021;51(4):775-85. Available from: [https://www.](https://www.sciencedirect.com/science/article/pii/S0049017221000615) [sciencedirect.com/science/article/pii/S0049017221000615](https://www.sciencedirect.com/science/article/pii/S0049017221000615).

Figure 1: Heterogeneity analysis of Placebo Response as Measured by the APS consdering subgroups based on APS Baseline values.

Figure 2:Heterogeneity analysis of Placebo Response as Measured by the PGA consdering subgroups based on PGA Baseline values.

Figure 3:Heterogeneity analysis of Placebo Response as Measured by the FIHOA consdering subgroups based on FIHOA Baseline values.
